# Machine learning-based decision support system for orthognathic diagnosis and treatment planning

**DOI:** 10.1186/s12903-024-04063-6

**Published:** 2024-02-28

**Authors:** Wen Du, Wenjun Bi, Yao Liu, Zhaokun Zhu, Yue Tai, En Luo

**Affiliations:** 1 State Key Laboratory of Oral Diseases & National Center for Stomatology & National Clinical Research Center for Oral Diseases, West China Hospital of Stomatology, Sichuan University, Chengdu, 610041 Sichuan China; 2 Department of Oral and Maxillofacial Surgery, Peking University School and Hospital of Stomatology & National Clinical Research Center for Oral Diseases & National Engineering Laboratory for Digital and Material Technology of Stomatology & Beijing Key Laboratory of Digital Stomatology, Beijing, China; 3 School of Electric Power Engineering, Nanjing Institute of Technology, Nanjing, China

**Keywords:** Artificial bee colony, Artificial intelligence, Dento-maxillofacial deformities, Diagnosis, Machine learning, Surgical plan

## Abstract

**Background:**

Dento-maxillofacial deformities are common problems. Orthodontic–orthognathic surgery is the primary treatment but accurate diagnosis and careful surgical planning are essential for optimum outcomes. This study aimed to establish and verify a machine learning–based decision support system for treatment of dento-maxillofacial malformations.

**Methods:**

Patients (*n* = 574) with dento-maxillofacial deformities undergoing spiral CT during January 2015 to August 2020 were enrolled to train diagnostic models based on five different machine learning algorithms; the diagnostic performances were compared with expert diagnoses. Accuracy, sensitivity, specificity, and area under the curve (AUC) were calculated. The adaptive artificial bee colony algorithm was employed to formulate the orthognathic surgical plan, and subsequently evaluated by maxillofacial surgeons in a cohort of 50 patients. The objective evaluation included the difference in bone position between the artificial intelligence (AI) generated and actual surgical plans for the patient, along with discrepancies in postoperative cephalometric analysis outcomes.

**Results:**

The binary relevance extreme gradient boosting model performed best, with diagnostic success rates > 90% for six different kinds of dento-maxillofacial deformities; the exception was maxillary overdevelopment (89.27%). AUC was > 0.88 for all diagnostic types. Median score for the surgical plans was 9, and was improved after human–computer interaction. There was no statistically significant difference between the actual and AI- groups.

**Conclusions:**

Machine learning algorithms are effective for diagnosis and surgical planning of dento-maxillofacial deformities and help improve diagnostic efficiency, especially in lower medical centers.

**Supplementary Information:**

The online version contains supplementary material available at 10.1186/s12903-024-04063-6.

## Introduction

Dento-maxillofacial deformities are a common problem in clinical practice, with approximately 5% of the population exhibiting abnormal jaw development [1, 2]. Orthodontic–orthognathic surgery is the primary treatment for these deformities, but accurate diagnosis and careful surgical planning are essential for optimum outcomes [3–5]. The diagnosis of dento-maxillofacial deformities and the design of orthognathic surgery rely primarily on clinical examination, assessment of occlusal relationship, and cephalometry [1].

Artificial intelligence (AI) has demonstrated the capability to identify skeletal malocclusions and predict the need for orthognathic surgery [6, 7]. However, previous studies have mainly relied on lateral cephalometric data, which inevitably results in information loss. These studies diagnosed dento-maxillofacial deformities through imaging, and specific surgical plans were not designed. Moreover, certain scholars have employed 3D point cloud deep learning to generate bone templates for guiding bone block movement schemes in orthognathic surgery [8]. While this represents a novel approach toward AI-assisted orthognathic surgery design, there is a lack of clinical validation for this method.

The present study created an interactive decision support system that could output an accurate diagnosis of dento-maxillofacial deformities and recommend individual surgical plans based on surgeon preferences.

## Methods

This study was conducted in the West China Hospital of Stomatology of Sichuan University and was approved by the Investigational Review Board of West China Hospital of Stomatology (WCHSIRB-OT-2019-125). All procedures were in accordance with relevant guidelines and regulations.

### Patients

A total of 574 patients who visited the Department of Orthognathic and Temporomandibular Joint Surgery, West China Hospital of Stomatology, Sichuan University, from January 2015 to August 2020 formed the training cohort. We excluded patients with missing teeth (other than third molars), previous history of orthodontic and orthognathic treatment, dento-maxillofacial deformities caused by fractures or tumors, and those who did not undergo digital surgical planning. A total of 89 patients were excluded.

Demographic information, extraoral and intraoral photographs, and pretreatment cephalometric measurements were extracted from the clinical records (Table 1) [2, 9], and 27 features were used as input features (Table S1). The input features were preprocessed to ensure that all were quantified before being used for model training. The diagnosis of dento-maxillofacial deformity was made by an orthognathic surgeon (Dr. Luo) with 19 years of clinical experience. The deformities included those of maxillary development, mandibular development, maxillary deviation, and mandibular deviation.

**Table 1:** Demographic and clinical characteristics of patients in this study

*N* = 574	Male			Female		
Age, years, mean (SD)	23.4 (7.2)			26.3 (8.5)		
Sex	203 (35.4%)			371 (64.6%)		
Maxillary development	Underdevelopment	201 (35%)	Normal	210 (36.6%)	Overdevelopment	163 (28.4%)
Maxillary deviation	Deviation		208 (36.2%)	Non-deviation		366 (63.8%)
Mandibular development	Underdevelopment	175 (30.5%)	Normal	138 (24%)	Overdevelopment	261 (45.5%)
Mandibular deviation	Deviation		253 (44.1%)	Non-deviation		321 (55.9%)

SD, standard deviation

### Diagnosis model

Different types of dento-maxillofacial deformities were diagnosed based on their characteristics, resulting in a comprehensive collection of 28 clinical features. These features were used to evaluate maxillary and mandibular development and deviations. The measurements were obtained with spiral computerized tomography (CT) utilizing the Mimics 16.0 software (Materialise Inc., Leuven, Belgium). All measurements were repeated twice and averaged by two orthognathic surgeons after an interval of at least 1 week. Cephalometric measurements indicating the degree of jaw deflection were considered positive from top left to bottom right.

Common classifier algorithms included extreme gradient boosting (XGBoost), discriminant analysis, naive Bayesian classification, neural networks, and support vector machines (SVM). These models were applied to the test set to evaluate the appropriateness and effectiveness of each model and determined the most appropriate and superior model. To ensure optimal classifier performance and generalizability, we randomly divided the data into training, validation, and test sets in a ratio of 7:3:1. The binary relevance extreme gradient boosting (BR-XGBoost) algorithm demonstrated superior performance compared to the remaining four machine learning algorithms in the validation set.

BR-XGBoost algorithm was used to process the data on dental and maxillofacial malformations and realize intelligent diagnosis [10]. Similar to the traditional supervised tree model algorithm based on the boosting principle, BR-XGBoost integrates several weak classifiers into a strong classifier through multiple rounds of iteration and residual fitting; the method has good generalization performance and operation efficiency. We modeled the diagnosis problem as *Q* single label two classification models. *Q* is the number of labels, and each label represents whether the patient has this particular kind of disease. For disease *j*, a training set ${D_j}=\left\{ {\left( {{X_i},{y_i}} \right)|1 \leqslant i \leqslant n} \right\}\left( {1 \leqslant j \leqslant Q} \right)$ is set up, where *i* is the sample serial number, *X* is the patient’s clinical symptom vector, and ${y_i} \in \left\{ {1,0} \right\}$ indicates whether sample *i* belongs to label *j*. The XGBoost binary classification model was constructed based on ${D_j}$ training, so that the prediction result ${y_j}$ of label *j* could be obtained. Then, multiple binary classifiers were combined into BR-XGBoost to output the multilabel diagnosis $Y=\left[ {{y_1},{y_2},...,{y_Q}} \right]$.

Considering the differences in patients’ clinical symptoms, it was necessary to study the generalization performance of the proposed algorithm. The feature selection of each $XGBoos{t_j}$ was based on the forward sequence selection method. For label *j*, first, the importance ranking of all features was obtained based on BR-XGBoost. The features with the top rankings were then added to the feature subset (initially empty set), and the cross-validation classification accuracy of the feature subset was calculated after each addition. If the classification accuracy was improved, the feature was retained, otherwise it was eliminated, and the optimal feature subset of label *j* was obtained by traversing all features. The performance of the model was not only affected by the training set, but also by the selection of its built-in parameters, that is, super parameters. To avoid the complexity and uncertainty of manual parameter adjustment, the distributed asynchronous hyper parameter optimization module on the Pycharm platform was used. Based on the Bayesian optimization theory, the cross-validation method was adopted to optimize each BR-XGBoost, and determine the range of hyper-parameters, thereby improving model accuracy.

To evaluate the performance of the artificial intelligence model, the following normal metrics were used.

1. Accuracy: $$Accuracy=\frac{{TP+TN}}{{TP+FP+TN+FN}}$$
2. Precision: $$Precision=\frac{{TP}}{{TP+FP}}$$
3. Recall: $$Recall=\frac{{TP}}{{TP+FN}}$$
4. Specificity: $$Specificity=\frac{{TN}}{{FP+TN}}$$
5. F1score: $$F1score=2 \cdot \frac{{Precision \cdot Recall}}{{Precision+Recall}}$$
6. AUC: Area under the curve.

where TP = true positive, TN = true negative, FP = false positive, and FN = false negative.

### Surgical design model

Following preoperative orthodontics, cephalometric measurements were re-performed to obtain patient-specific characteristics. The terminal occlusal relationship was recorded by the 3D spatial relationship of three points marked on the maxilla and the mandible. The reference intervals of these characteristics were established based on their normal intervals in the Chinese population and our clinical experience. Six sets of data were used to accurately describe the 3D movement of the maxilla, mandible, and chin according to the commonly used clinical methods.

The AI-based surgical model employed the adaptive artificial bee colony (aABC) algorithm to calculate the maxillary movement, mandibular movement, and mentum movement. The 3D translation and rotation of each part in the surgical scheme constitute the solution space of the aABC algorithm. All possible solutions in the solution space are expressed by honey source, and the degree of honey source is measured by the value of fitness function. Bees can be divided into three types according to division of labor: collecting bees, following bees, and reconnaissance bees (collecting bees and following bees each account for half of the total number of bees; the collecting bees corresponding to inferior honey sources are transformed into reconnaissance bees to search for new honey sources). The specific search for honey source is as follows:

1. The algorithm was initialized.
2. Algorithm optimization objectives were determined. In this step, the reasonable range of values for each feature was determined. These ranges were superimposed to form the optimized feasible region of the algorithm proposed in this paper. The optimal eigenvalue within the feasible range was determined as the final surgical plan. This study assumed that feature values closer to the middle of the range were better.
3. Intelligent exploration of the optimal solution for surgical plans based on aABC algorithm: With increase of the number of cycles of the algorithm, the weight between algorithm exploration and development constantly changes. When the cycle starts, the algorithm has a high weight in exploration, and the global search ability is strengthened, so it is not easy to fall into local optimization. The continuous increase in the number of cycles gradually decreases the adaptive coefficient value. At this time, the algorithm tends to develop the region guided by the global optimal solution, which improves the convergence speed and accuracy of the algorithm.
4. For the following bee, the calculated probability value selects the honey source and carries out neighborhood search to generate a new solution, and selects the honey source with better fitness value.
5. If no better honey source is found within a limited number of times, the honey source is discarded and a new honey source is randomly generated.
6. The optimal honey source (global optimal solution) found by all bees is saved, and the termination condition of the algorithm (maximum number of iterations) is judged. If the conditions are met, the optimal surgical scheme is returned and the algorithm is terminated. Otherwise, we return to the first step to continue the algorithm.

The surgical plan generated by the surgical design model could be adjusted according to expert preferences, a process called human-computer interaction. The surgical plans were generated using features of the normal population. In case of dissatisfaction with the current design plan regarding the feasibility and outcome of the operation, the surgeon could propose potential modifications to the jaw’s movement.

The AI-generated surgical plan was subjectively and objectively evaluated using the test set. The subjective evaluation of the surgical plan encompassed both feasibility and effectiveness assessments, conducted by three experienced orthognathic surgeons from our center. None of these surgeons were involved in the study’s design, and only evaluated the effect of the surgical protocol based on their clinical experience. Prior to commencing the study, intraclass correlation coefficients (ICCs) for the realistic surgical plan from the test set were analyzed among the three participating surgeons by the realistic surgical plan from the test set. The objective evaluation included the difference in bone position between the AI-generated and actual surgical plans, as well as discrepancies in postoperative cephalometric analysis outcomes. Measurements were conducted using 3-matic 9.0 software (Materialise, Belgium). Paired t test was used to analyze statistical differences in cephalometric results.

**Fig. 1:**
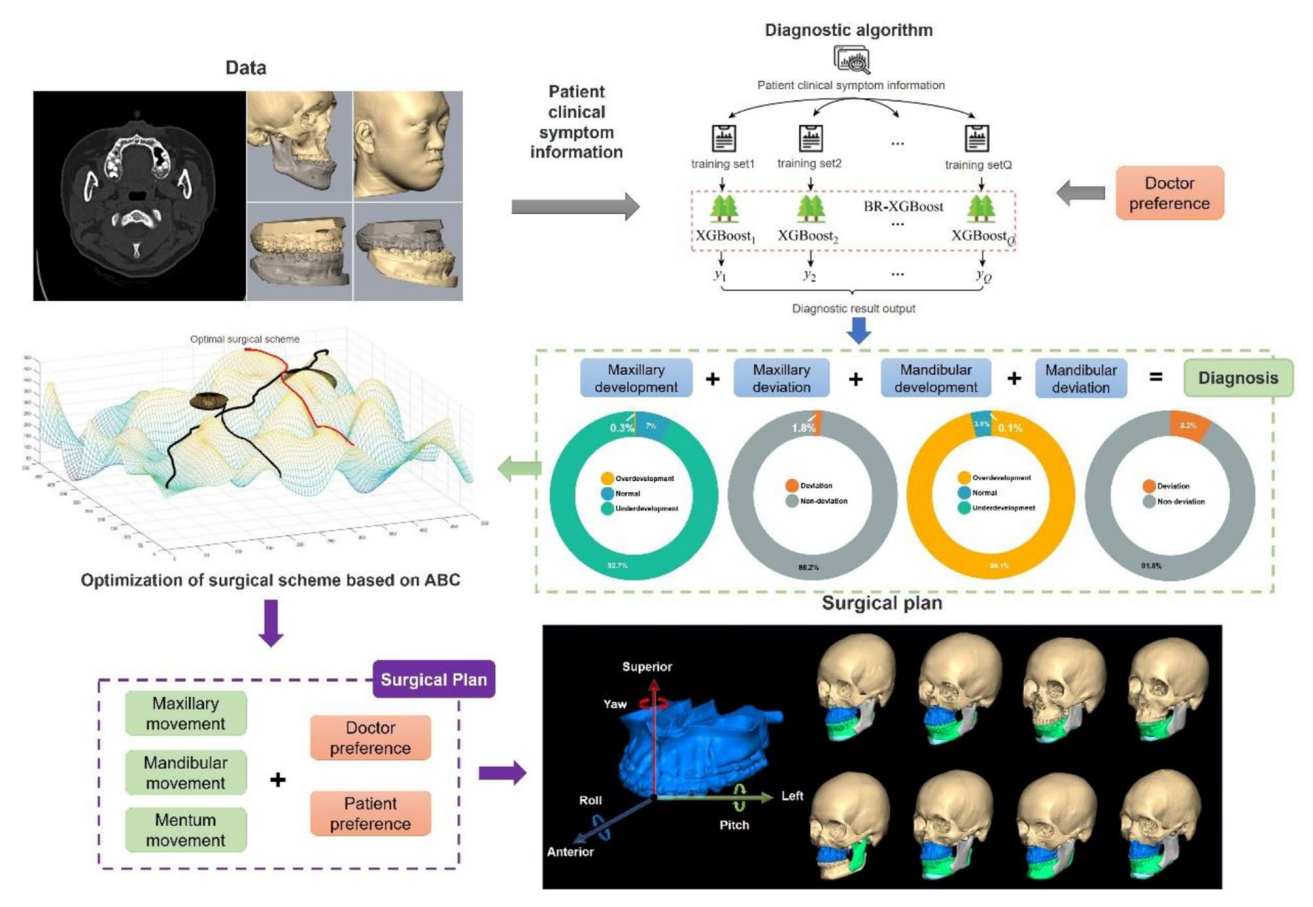
Pipeline of the proposed decision support system for orthognathic diagnosis and treatment planning

Figure 1 presents an overview of the decision support system and treatment planning.

## Results

### Performance of diagnostic system

**Fig. 2:**
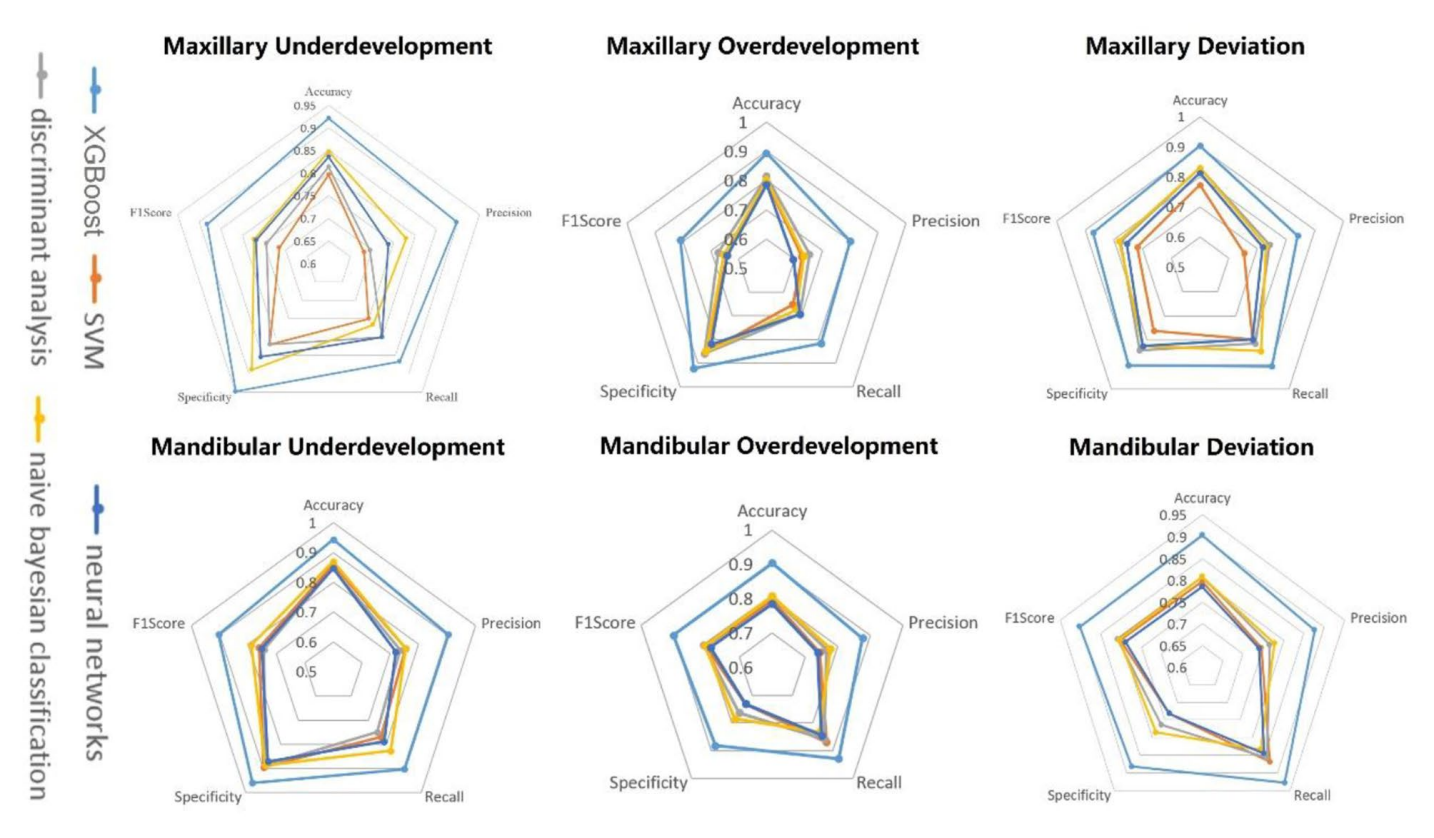
The performance of five machine learning algorithms (BR-XGBoost, SVM, neural network, discriminant analysis, Bayesian classification) was evaluated in six classification problems related to dento-maxillofacial deformities: maxillary overgrowth, maxillary undergrowth, maxillary deviation, mandibular overgrowth, mandibular undergrowth, and mandibular deviation. The radar chart shows the algorithm performs on Accuracy, Precision, Recall, Specificity, and F1 score. The BR-XGBoost algorithm consistently demonstrated superior results across all six classification problems

**Fig. 3:**
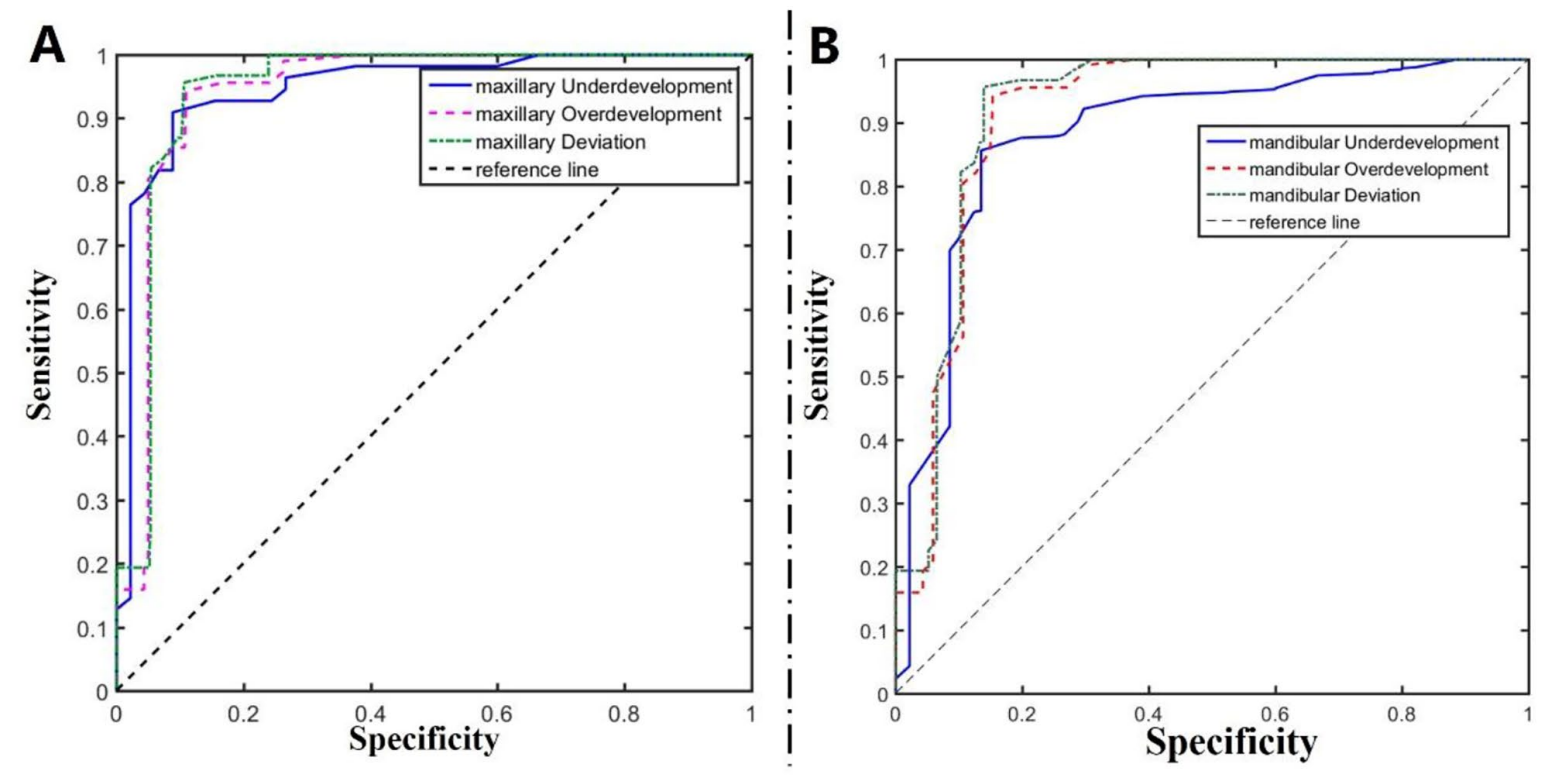
ROC curve of the BR-XGBoost algorithm in the diagnosis of dento-maxillofacial deformities. The AUC values ranged from 0.881 to 0.982 in different diagnostic classification tasks

Of the five different types of machine learning algorithms, the BR-XGBoost algorithm showed the highest accuracy and sensitivity for classification of different types of dento-maxillofacial deformities (Fig. 2). The receiver operating characteristic (ROC) curve in Fig. 3 illustrates the performance of the BR-XGBoost algorithm in the diagnosis dento-maxillofacial deformities. The AUC for the model ranged from 0.881 to 0.982. The best performance was for diagnosis of deviation, followed by mandibular overdevelopment and maxillary underdevelopment. The diagnostic model classified the dento-maxillofacial deformities, and the combination of the two provided the final diagnosis.

### Performance of the surgical design model

**Fig. 4:**
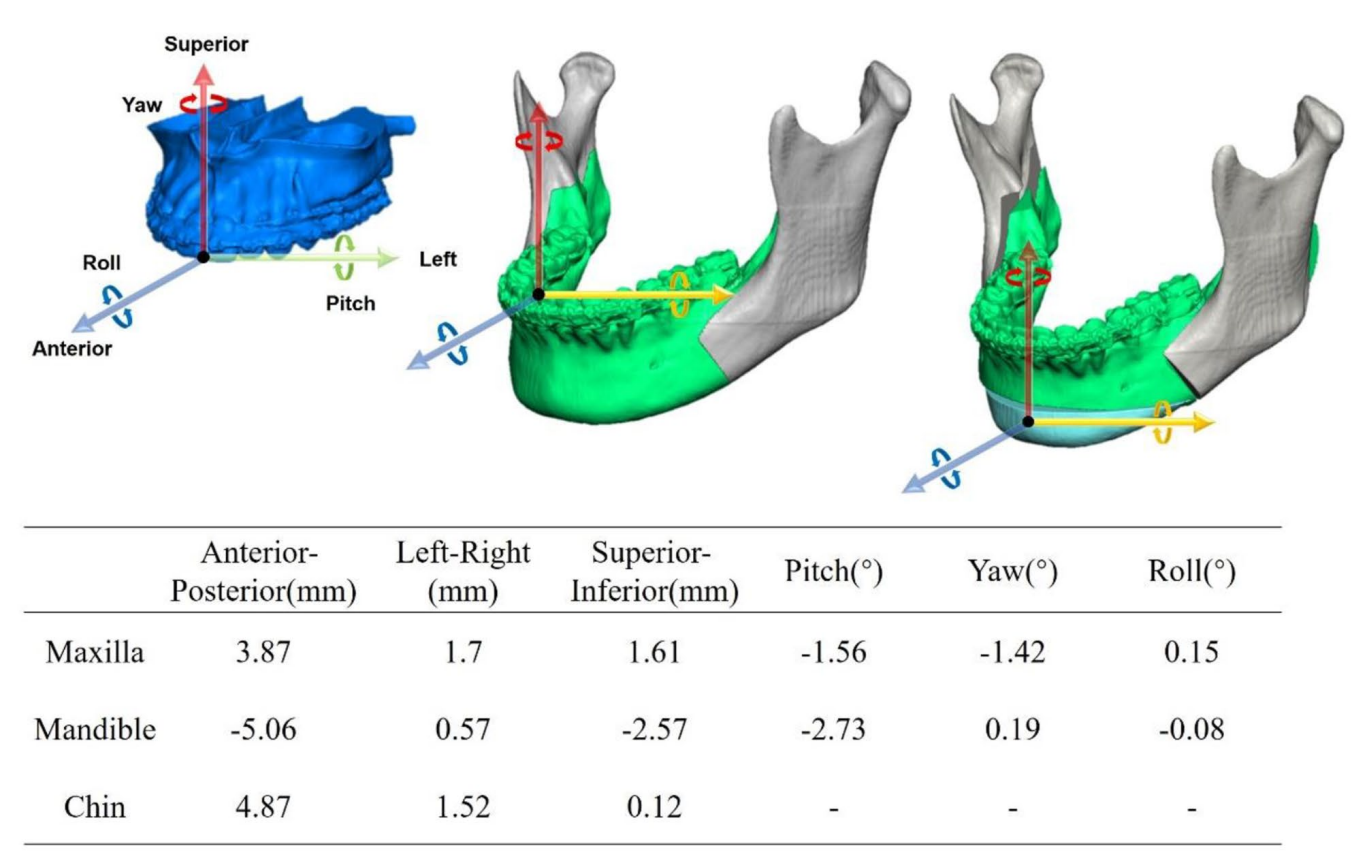
Personalized surgical plan designed by the system for a skeletal class III malocclusion patient

The system can output a personalized surgical plan (Fig. 4). The output results contain six 3D parameters representing rotation and movement of maxilla, mandible, and chin. For movement of maxilla and mandible, the corresponding incisor point was taken as the movement center. For the chin, the pogonion point was taken as the movement center and is reduced to have translational movement only.

The ICCs for the three participating orthognathic surgeons in this study, are presented in Table S2. The effectiveness and feasibility of intelligently designed surgical procedures were rated on a scale of 1 to 10. The surgical plans were individually evaluated by the three surgeons; the distribution of their scores is presented in Table 2.

**Table 2:** Evaluation of surgical effect and feasibility

**Effect of surgery**												
Score	1 (Unsatisfactory)	2	3	4	5	6	7	8	9	10 (Satisfactory)	Median	Mean ± SD
Preliminary plan	0	0	0	0	6	3	9	21	69	42	9	8.8 ± 1.21
Revised plan	0	0	0	0	0	3	3	36	45	63	9	9.08 ± 0.97
**Surgical feasibility**												
Score	1 (Unsatisfactory)	2	3	4	5	6	7	8	9	10 (Satisfactory)	Median	Mean ± SD
Preliminary plan	0	0	3	0	9	9	18	33	27	51	9	8.34 ± 1.69
Revised plan	0	0	0	0	0	3	18	30	36	63	9	8.92 ± 1.14

**Fig. 5:**
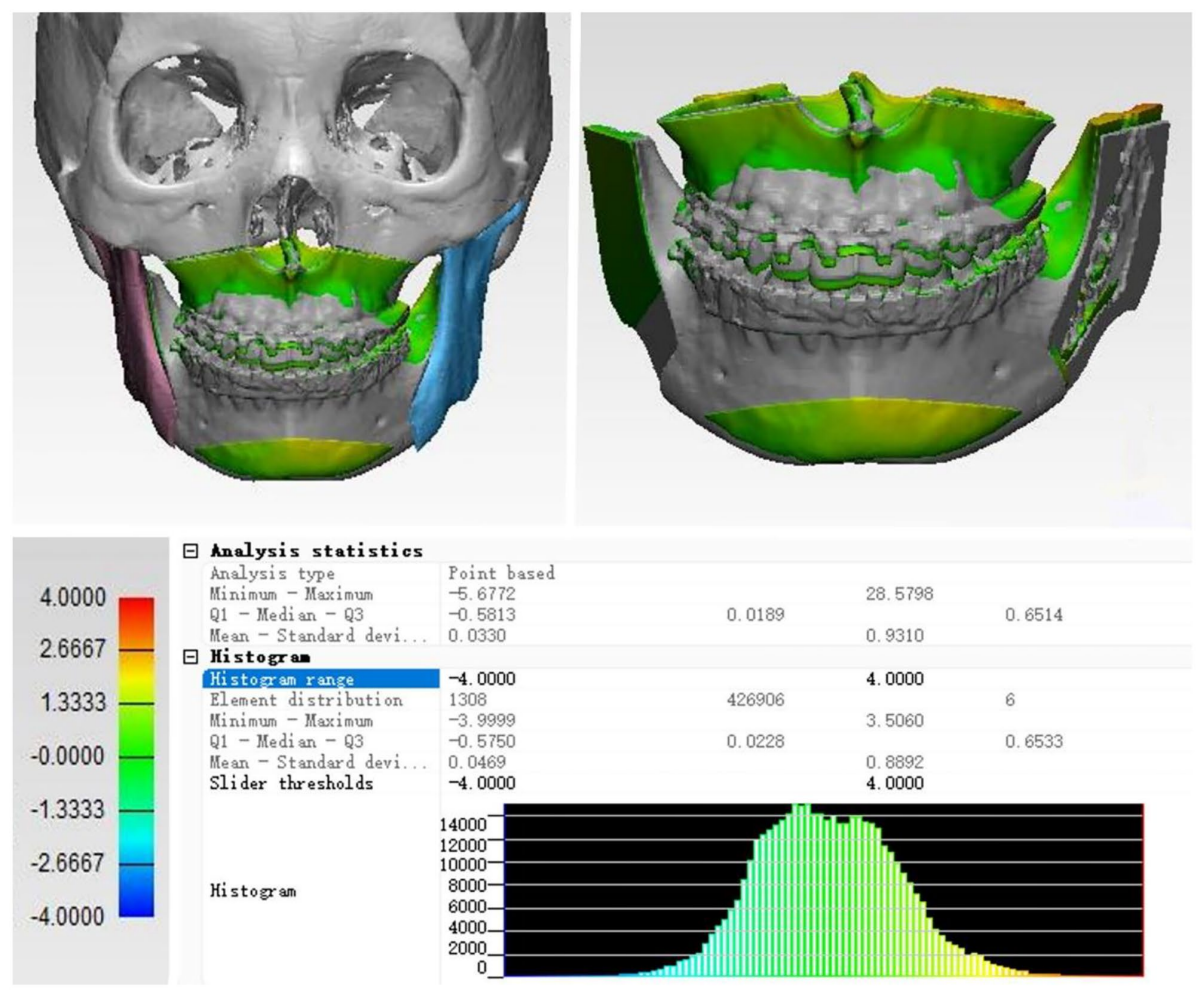
A color distance map was applied to align with the two surgical plans, where the colored bone segment depicted represents the actual surgical plan. The difference distribution is shown in the lower right corner

The fitting analysis process is shown in Fig. 5, while the discrepancies in the maxilla, mandible, and chin positions under two distinct schemes are presented in Table 3.

**Table 3:** Deviations between the surgical design model plans and the actual surgeries

	Average deviation (mm)	SD (mm)	Error < 2 mm (%)	Error < 3 mm (%)	Error < 4 mm (%)
Maxilla	2.15	1.24	48.1	80.7	95.8
Mandible	2.81	1.57	31.9	71.5	91.4
Chin	3.32	1.79	24.8	59.8	78.6

The differences in three-dimensional cephalometric measurements result between the actual surgical and the AI-model are presented in Table 4.

**Table 4:** Differences in cephalometric measurements between different plans after simulation

Cephalometric values	Actual plan	AI plan
	Mean ± SD	Mean ± SD
Or-U6(R)/Or-U6(L) (%)	99.6 ± 1.3	100.1 ± 1
OP-FHP(°)(Roll)	-0.3 ± 0.5	0.1 ± 0.3
Go-Me (R)/ Go-Me (L) (%)	99.6 ± 1.2	99.5 ± 0.9
Pog-MSP (mm)	0.15 ± 0.7	-0.23 ± 0.2
OP-FHP (°)(Pitch)	10.1 ± 2.4	12.6 ± 1.2
SNA (°)	80.4 ± 2.5	81.5 ± 1.3
SNB (°)	79.6 ± 2.2	79.3 ± 1.5
ANB(°)	2.0 ± 1.3	2.6 ± 0.6
SNPog(°)	78.6 ± 2.3	80.5 ± 1.6
N-ANS/ANS-Me (%)	82.4 ± 2	82.7 ± 1.2

There were no statistically significant differences between the two groups

**Fig. 6:**
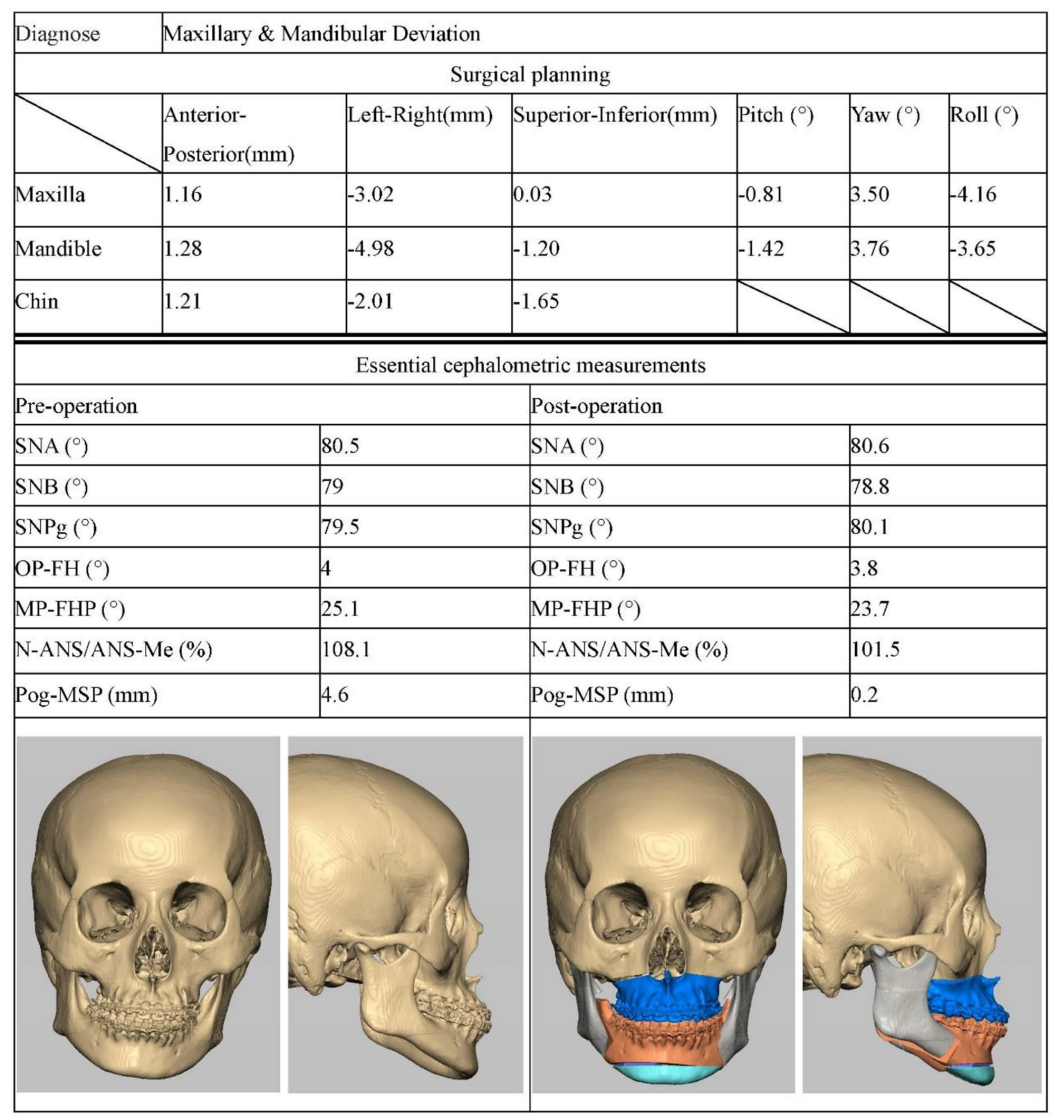
Representative case A. The decision support system diagnosed the patient with maxillary and mandibular deviation and designed a surgical plan. Three-dimensional visualization of the surgical effect was processed using Freeform plus software (3DSystems Inc., Rock Hill, USA). Lefort I osteotomy, BSSRO, and genioplasty were performed through the virtual surgical method. After completion of the osteotomy, the bone block was moved according to the AI-generated surgical plan. The patient’s dento-maxillofacial deformity improved significantly and the cephalometric measurements returned to normal

Figure 6, and 7 present the cephalometric data and automatically generated surgical plans for three patients with dento-maxillofacial deformities.

**Fig. 7:**
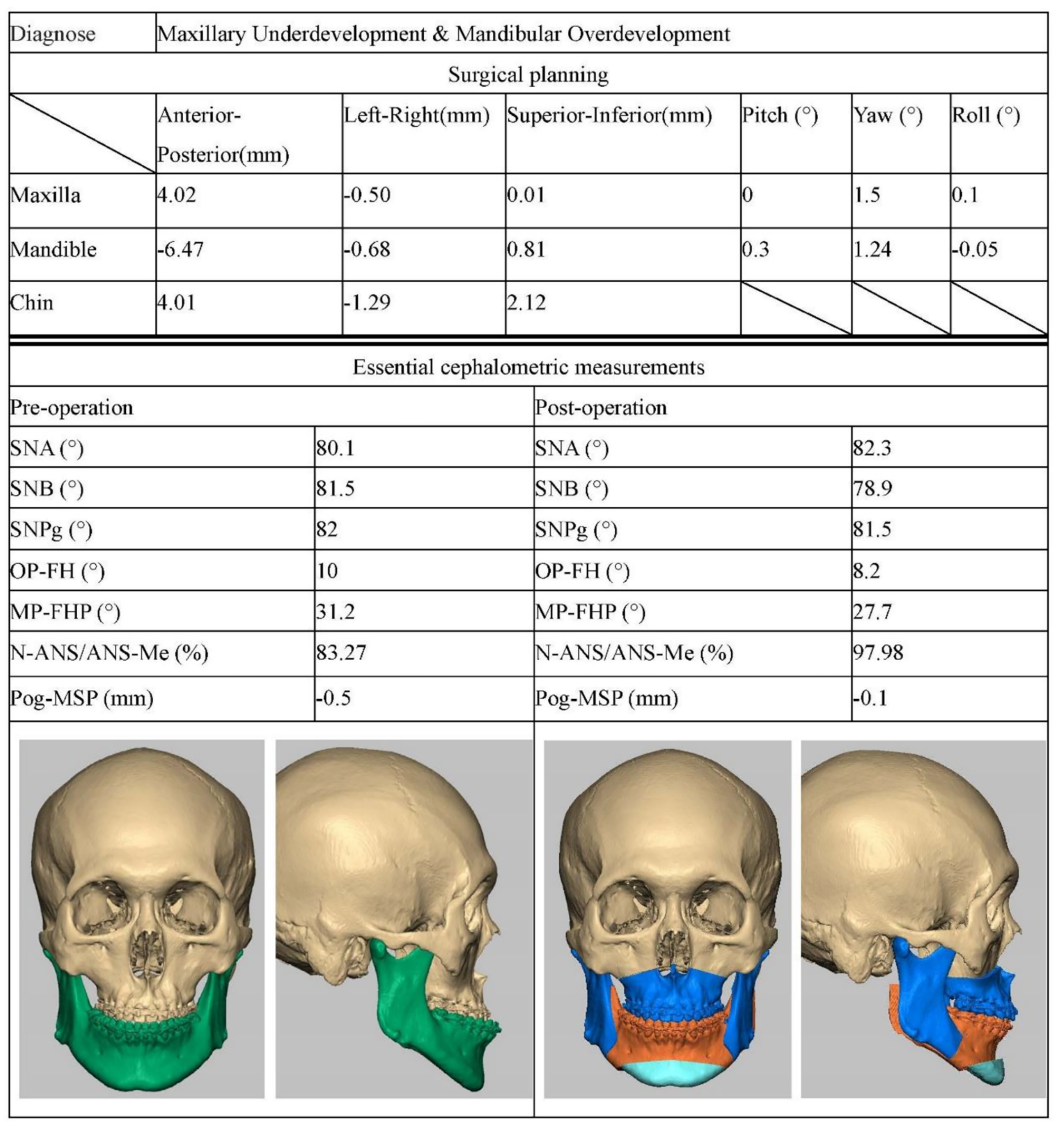
Representative case B. The patient was diagnosed with maxillary underdevelopment and mandibular overdevelopment by the decision support system. Three rounds of interaction between the surgeons and the system resulted in the final operation plan. Three-dimensional visualization of the surgical effect was processed using Freeform plus software (3DSystems Inc., Rock Hill, USA). Lefort I osteotomy, BSSRO, and genioplasty were performed through the virtual surgical method. After completion of the osteotomy, the bone block was moved according to the AI-generated surgical plan. The patient’s dento-maxillofacial deformity improved significantly and the cephalometric measurements returned to normal

## Discussion

In this study, we propose a machine learning–based decision support system for orthognathic diagnosis and treatment planning. The system can diagnose dento-maxillofacial deformities and output an interactive and personalized surgical option by analyzing patients’ clinical features and the preferences of both the surgeon and the patient. The system achieved an overall accuracy rate of > 90% and was highly rated by expert reviewers.

The diagnosis and treatment planning of dento-maxillofacial deformities is a multifaceted process influenced by various factors, including ethnicity, esthetics, and diagnostic practices [2, 11]. The surgeon’s experience also plays an important role. Dento-maxillofacial deformities manifest as an incongruity in the spatial relationship between the teeth and the jaw, and generally requires orthognathic and orthodontic treatment. Advancements in orthognathic surgery have led to the development of several objective indicators for aiding the surgeons in diagnosis and surgical planning. The classical two-dimensional cephalometry, model surgery, and VTO techniques can provide surgeons with valuable quantitative diagnostic information and aid meticulous surgical planning [12]. With the advancements in computer technology, digital surgical technology has enabled the surgeons to perform computerized preoperative osteotomy and postoperative bone morphology simulations [13, 14]. This holds great significance for the precise design and implementation of the surgical plan.

Digital surgery has become standard practice in orthognathic surgery across numerous clinical centers. Its execution requires a collaboration among professional institutions, their affiliated hospitals, and digital surgery design companies. However, certain challenges persist, including a complex manual operation process, a significant need for repetitive labor, low efficiency, and prolonged time consumption [15]. The diagnosis and surgical design of dento-maxillofacial deformities adhere to highly standardized protocols, ensuring stability in the selection of surgical methods and enabling quantifiable surgical planning. These favorable conditions facilitate intelligent diagnosis and surgical design for dento-maxillofacial deformities. Considering the characteristics of orthognathic surgery, broader applications of AI in this domain are inevitable in the future. This may enhance the work efficiency, diagnostic accuracy, and treatment outcomes, thereby benefiting both doctors and patients.

AI can be used to accurately classify patients’ skeletal patterns, enabling the identification of cranial structures with varying degrees of developmental deviations. This evaluation can be conducted using cephalometric images to classify anteroposterior and vertical skeletal discrepancies [16, 17]. The combination of posteroanterior radiographs and lateral cephalograms can enhance accuracy and provide additional information on asymmetry [6, 18]. Previous studies have utilized AI to elucidate the necessity of orthognathic surgery. These studies utilized cephalometric parameters either independently or in conjunction with other assessments such as anteroposterior radiographs, occlusal models, and demographic variables for evaluating the need for orthognathic surgery [6, 17, 19].

The majority of existing studies have primarily focused on the identification of patients requiring orthognathic surgery. While one study has utilized deep learning to generate a virtual jaw shape and employed this model as a reference for designing specific jaw movements, the constructed model requires validation before clinical application [8]. To the best of our knowledge, no study has yet achieved the successful development of personalized and precise orthognathic surgical design tailored specifically to individual patients.

The XGBoost algorithm has unique advantages in dealing with diagnostic problems: it is excellent for parallel operation and can run large-scale data quickly; it can automatically optimize split nodes and is good at dealing with irregular data with many outliers and missing values; and, finally, it is interpretable and flexible [20, 21]. Further, the XGBoost algorithm can handle difficult over fitting, has higher accuracy of loss function solution, supports sparse data processing, and so on. Compared with the sample data in the industrial field where artificial intelligence is widely used, the sample size for the maxillofacial diagnosis problem studied is small and so is ideally suited for XGBoost, which is less demanding in terms of sample size. In this study, BR-XGBoost achieved the best overall performance, with overall accuracy rate of over 90%. Considering that some patient data were collected manually, there was a certain amount of deviation noise. We found that the proposed algorithm had good detection performance when the deviation conformed to the normal distribution. The clinical features exhibited variations between genders, with different reference intervals selected for different genders in this study [22].

The purpose of the orthognathic surgery was to correct incongruous three-dimensional positional relationships between the teeth and the jaws. Cephalometric indicators were the main clinical measures utilized for characterizing disharmony. The designed plan used several cephalometric indicators that are commonly used in the clinic as a basis for correcting the abnormalities. When designing the optimal feature target value for AI algorithms in this study, we believe that the best feature values were those closest to the middle of each feature range. The aABC algorithm is a bionics adaptive artificial intelligence technique for solving extremum problems [23, 24]. It has natural advantages in solving function optimization problems and is also the most applied algorithm in the field though there are no reports of its application in the medical field. This algorithm can be used to solve multivariable function problems. To maximize the efficiency of the algorithm, we chose to use the aABC [25, 26], which can adaptively change the weight of each influencing factor in the search equation to simultaneously provide good exploration and development ability. The aABC algorithm increases the operation speed by about 30%. In this study, single operation time for one set of data was ∼ 30 s.

The calculation of this process is complicated, and there may be multiple schemes that could meet the requirements. For cases requiring personalized surgical plans, this could be achieved by adjusting the optimal target feature values, a process called interactivity. The personalized parameter adjustment occurs in the 4) step of the aABC algorithm. Table 2 illustrates that the distribution of feasibility and effectiveness scores for the adjusted surgical protocol was superior to that for the primary output, indicating an improved effect of the surgical plan design.

It should be noted that cephalometric measurement is only one of the methods to evaluate dento-maxillofacial deformities, and using only cephalometric measurement to evaluate maxillofacial deformity will inevitably reduce diagnostic accuracy. However, the diagnostic accuracy rate in this study was acceptable. Except for mandibular underdevelopment, AUC was > 0.9 for all diagnostic problems. The AUC of mandibular underdevelopment was 0.881. The overall diagnostic accuracy for dento-maxillofacial deformities was over 90%. In the present study, the BR-XGBoost algorithm achieved the best overall performance. Our classification was more extensive and accurate compared to similar previous studies.

The surgical effect refers to the impact of the surgical plan on improving the patient’s dento-maxillofacial deformity, including profile and symmetry improvements. The subjective effectiveness mean score was 8.8, which further increased to 9.08 after human-computer interaction. Overall scores ranged from 8 to 10, with a median of 9, indicating that the AI-assisted surgical plan could successfully enhance facial appearance and achieve a high score deemed acceptable by experts.

Surgical feasibility refers to whether the extent of bone segment movement surpasses the surgical threshold, post-implementation jaw stability, and the risk of recurrence (e.g., the presence of bone contact). Subjective feasibility evaluation results indicated an initial average score of 8.34, which increased to 8.92 following human-computer interaction. Overall scores ranged from 7 to 10, with a median of 9, highlighting the clinical feasibility of AI-assisted surgical plans. Subjective evaluations revealed generally acceptable surgical outcomes. Prior to scoring, a consensus recommendation was reached by the three participating surgeons involved in the study. A higher ICC value, closer to 1, indicated a stronger level of agreement. The consistency test conducted on the three study demonstrated a high level of agreement in their evaluations.

A comparison of the positional deviation of the bone segments in the two distinct plans demonstrated that the average discrepancy in both maxilla and mandible was approximately 2 mm, with most regions exhibiting a deviation of < 4 mm. This indicates that there existed a certain level of disparity in jaw bone positioning between the two plans. However, considering the intricate three-dimensional morphology of the bone segment itself, no significant divergence was observed in subjective assessment scores for efficacy within either group. Hence, it can be inferred that the maxillary and mandibular movement plans fell within an acceptable range. A comparison of the measurement items between the two schemes revealed no statistically significant differences between the two groups of data for each item. Additionally, the changes in the measurement indices closely aligned with the normal range, indicating an improvement in jaw morphology and facial shape for the patients. As this experiment included individuals with both skeletal Class II and skeletal Class III malocclusions, there were no significant differences between the mean preoperative and postoperative values. However, compared to the preoperative cephalometric results, a significant reduction in the standard deviation was observed, suggesting a greater number of data points trending toward the normal range.

The proposed system also had some limitations. The prediction of post-orthognathic surgery soft tissue changes has long been a clinical challenge awaiting resolution. An intricate interplay of numerous factors significantly influences these changes. This study focused exclusively on hard tissue alterations and did not encompass the intricacies associated with soft tissue considerations [27]. In the future, it may become possible to take these factors into consideration and plan orthognathic-orthodontic therapy at the time of initial diagnosis [28].

In this study, jaw development was categorized into three conditions: overdevelopment, underdevelopment, and normal. However, this classification method lacks clinical information regarding the specific direction of jaw growth, making it difficult to determine whether a patient has a sagittal or vertical developmental issue, or their combination. The limited number of machine learning samples restricted this classification approach. A total of 574 patients were included in the study, with certain clinical patient types underrepresented due to data bias. To address this limitation in future studies, it is recommended to supplement the dataset with a wider range of patient types.

## Conclusions

This study developed an interactive decision support system capable of providing precise diagnoses for dento-maxillofacial deformities and offering personalized surgical plans. For this purpose, we employed BR-XGBoost and the aABC algorithm. The decision support system utilized a combination of objective evaluation indices and subjective assessment scores to accurately diagnose dento-maxillofacial deformities and generate orthognathic surgery plans aligning with clinical requirements. Moreover, the surgical plan could be customized based on expert preferences to cater to individual needs. Future validation of this decision support system requires the involvement of diverse clinical centers and a wider scope, subsequently extending its application to subordinate hospitals for providing decision support to young doctors. Furthermore, there is a need for further research on AI-assisted soft tissue prediction in orthognathic surgery, which could be combined with real-time soft tissue change predictions to improve the design of jaw surgery plans.

### Electronic supplementary material

Below is the link to the electronic supplementary material.

Supplementary Material 1

## Data Availability

The datasets used and/or analyzed during the current study are available from the corresponding author on reasonable request.

## Abbreviations

CT: Computerized tomography
AUC: Area under the curve
BR-XGBoost: The binary relevance extreme gradient boosting algorithm
TP: True positive
TN: True negative
FP: False positive
FN: False negative
aABC: Adaptive artificial bee colony
ROC: The receiver operating characteristic
AI: Artificial intelligence
SD: Standard deviation
