# Supplementary material for: Machine learning-based decision support system for orthognathic diagnosis and treatment planning
Source: BMC Oral Health. 2024 Feb 28;24:286. doi: 10.1186/s12903-024-04063-6 (PMC10902963; doi:10.1186/s12903-024-04063-6)
Supplement: Supplementary file 1 — Supplementary Material 1 [file 12903_2024_4063_MOESM1_ESM.docx]

**Table S1 Descriptions of the 27 features utilized for diagnosing dento-maxillofacial deformities.**

| **Clinical Significance** | **Features** | **Unis** | **Description** |
| --- | --- | --- | --- |
|  | Gender | - | Females are 0 and males are 1. |
| Maxillary development | SNA | ° | Reflcting relationship of the maxilla to the cranial base. |
|  | N-A(//FHP) | mm | Reflcting relationship of the maxilla with the nasal root |
|  | ANB | ° | Indicating the magnitude of the skeletal jaw discrepancy. |
|  | N-ANS/ANS-Me | % | Ratio of midface height to lower face height |
|  | OP-FHP | ° | Angle between the occlusal plane and the Frankfurt horizontal plane |
|  | U1-Stms | mm | Static teeth exposure |
|  | 1/-PP | ° | The intersection of the long axis of the upper central incisor and the palate plane |
|  | Nasolabial angle | ° | Nasolabial angle |
|  | Ls-EP | mm | The distance of upper lip to aesthetic plane. |
|  | IIA | ° | Angle between line through long axis of upper incisor and lower incisor |
|  | Overbite | mm | The vertical distance between the edges of upper and lower incisors. |
|  | Overjet | mm | The projection distance of the upper and lower central incisors' cutting edges on the occlusal plane. |
|  |  |  |  |
| Mandibular development | SNB | ° | Reflcting relationship of the mandible to the cranial base. |
|  | N-B(//FHP) (mm) | mm | Reflcting relationship of the mandibular with the nasal root |
|  | N-Pog(//FHP) (mm) | mm | Reflcting relationship of the chin with the nasal root |
|  | Sn-Stms/Stms-Me’ | % | Ratio of lower one-third face height |
|  | MP-FHP (°) | ° | Mandibular plane angle |
|  | /1-MP | ° | The intersection of the long axis of the lower central incisor and the mandibular plane |
|  | Si-LiPog’ | mm | The depth of Mentolabial sulcus |
|  | Li-EP | mm | The distance of lower lip to aesthetic plane. |
|  |  |  |  |
| Maxillary deviation | Or(R)-U6(R)/Or(L)-U6(L) | % | Reflecting the asymmetrical of maxilla in vertica |
|  | U6(R)Z/ U6(L)Z | % | Reflecting the asymmetrical of maxilla in sagittal |
|  | U6(R)-Palate/U6(L)-Palate | % | Reflecting the asymmetrical of maxilla in vertical |
|  |  |  |  |
| Mandibular deviation | Go-Pog(R)/Go-Pog(L) | % | Mandibular body symmetry on both sides |
|  | Co-Go(R)/Co-Go(L) | % | Mandibular ramus symmetry on both sides |
|  | Pog-MSP (mm) | mm | Deflection of the mentum |

**Table S2 The ICC of three participating orthognathic surgeons for evaluation of surgical plan.**

|  | Intraclass Correlation Coefficient |
| --- | --- |
| Evaluation of surgical effect | 0.82 |
| Evaluation of surgical feasibility | 0.94 |
